# Quantitative Proteomic Analysis of Niemann-Pick Disease, Type C1 Cerebellum Identifies Protein Biomarkers and Provides Pathological Insight

**DOI:** 10.1371/journal.pone.0047845

**Published:** 2012-10-29

**Authors:** Stephanie M. Cologna, Xiao-Sheng Jiang, Peter S. Backlund, Celine V. M. Cluzeau, Michelle K. Dail, Nicole M. Yanjanin, Stephan Siebel, Cynthia L. Toth, Hyun-sik Jun, Christopher A. Wassif, Alfred L. Yergey, Forbes D. Porter

**Affiliations:** 1 Program in Developmental Endocrinology and Genetics, National Institute of Child Health and Human Development, National Institutes of Health, Department of Health and Human Services, Bethesda, Maryland, United States of America; 2 Biomedical Mass Spectrometry Facility, National Institute of Child Health and Human Development, National Institutes of Health, Department of Health and Human Services, Bethesda, Maryland, United States of America; Mental Health Research Institute of Victoria, Australia

## Abstract

Niemann-Pick disease, type C1 (NPC1) is a fatal, neurodegenerative disorder for which there is no definitive therapy. In NPC1, a pathological cascade including neuroinflammation, oxidative stress and neuronal apoptosis likely contribute to the clinical phenotype. While the genetic cause of NPC1 is known, we sought to gain a further understanding into the pathophysiology by identifying differentially expressed proteins in *Npc1* mutant mouse cerebella. Using two-dimensional gel electrophoresis and mass spectrometry, 77 differentially expressed proteins were identified in *Npc1* mutant mice cerebella compared to controls. These include proteins involved in glucose metabolism, detoxification/oxidative stress and Alzheimer disease-related proteins. Furthermore, members of the fatty acid binding protein family, including FABP3, FABP5 and FABP7, were found to have altered expression in the *Npc1* mutant cerebellum relative to control. Translating our findings from the murine model to patients, we confirm altered expression of glutathione s-transferase α, superoxide dismutase, and FABP3 in cerebrospinal fluid of NPC1 patients relative to pediatric controls. A subset of NPC1 patients on miglustat, a glycosphingolipid synthesis inhibitor, showed significantly decreased levels of FABP3 compared to patients not on miglustat therapy. This study provides an initial report of dysregulated proteins in NPC1 which will assist with further investigation of NPC1 pathology and facilitate implementation of therapeutic trials.

## Introduction

Niemann-Pick disease, type C (NPC) is a fatal, neurodegenerative disorder characterized by mutations of either *NPC1* or *NPC2*. Decreased function of either protein results in impaired cholesterol transport out of the late endosomal/lysosomal system and therefore accumulation of unesterified cholesterol and glycosphingolipids [as reviewed in [1]–[3]]. Secondarily, a cascade of pathological events occurs including insufficient oxysterol production [4], changes in intracellular calcium homeostasis [5], [6], neuroinflammation [7], apoptosis [8] and oxidative stress [9]–[13]. The clinical spectrum of NPC1 is broad. Clinical symptoms and age of onset are heterogeneous and progression appears linear after onset of symptoms and occurs over years [14]. These factors complicate the development of therapeutic interventions. Common clinical symptoms include neonatal liver dysfunction [15], and neurological symptoms including cerebellar ataxia, seizures and supranuclear vertical gaze palsy [1]. To date, a universally approved therapy for NPC1 is lacking. A randomized trial using miglustat, (N-butyldeoxynojirimycin, Zavesca®) a glycosphingolipid synthesis inhibitor, showed improvement of neurological symptoms after one year; however, long-term efficacy has yet to be established [16]–[18]. Although approved by the European Medicines Agency (EMA), miglustat has not been approved by the FDA for use in NPC1. Recent data evaluating the administration of 2-hydroxypropyl-beta-cyclodextrin (HP-β-CD) in animal model studies suggests that HP-β-CD may be a potential therapy for NPC1 that needs to be investigated [19], [20].

The neurological phenotype of the *Npc1* null mouse model replicates the disease process seen in humans. *Npc1* mutant mice are non-symptomatic from birth until approximately 5 weeks of age. Tremors are observed at 5 weeks followed by ataxia at 7–9 weeks. *Npc1* mutant mice are terminal by 11–12 weeks of age.

Neurodegeneration is a major aspect of NPC1. Specifically, Purkinje cell degeneration onset is early and extensive [21] however neurodegeneration is not limited to Purkinje cells. Ong and coworkers [22] described significant degeneration in the thalamus, hypothalamus, globus pallidus, midbrain, pons and medulla oblongata. Recently the thalamus has also been reported to be significantly affected early in *Npc1* mice [23]. In an attempt to further understand neurodegeneration that occurs in NPC1, Loftus *et al.*, generated a prion promotor-driven transgenic mouse in which many of the NPC1 phenotypes were reversed including prevention of Purkinje cell loss [24]. Recent reports suggest that cerebellar Purkinje death in NPC1 is a cell autonomous process [25], [26]. Studies in which *Npc1* was deleted from neurons result in similar phenotypes of mice with global deletions; however, removal of *Npc1* specifically in astrocytes of mice resulted in no discernable Niemann-Pick disease, type C1 phenotype and controlled deletion of *Npc1* at 6 weeks of age did not alter the progression or lifespan from the time the deletion occurred [27]. Similarities in the neurodegenerative features of NPC1 and Alzheimer disease (AD) have also been noted [11]. These include presence of neurofibrillary tangles, increased beta-amyloid in cerebrospinal fluid (CSF) [28], hyperphosphorylation of tau protein [29], and increased activity of cyclin-dependent kinase 5 [30].

Biomarkers, specifically protein biomarkers, can provide insight into the pathological processes contributing to disease progression. As such, they also provide tools to facilitate the development of therapeutic interventions. For this purpose, protein biomarkers can be either disease specific or reflect nonspecific pathological processes contributing to neurodegeneration. To date, a comprehensive series of biological markers or surrogates corresponding to different aspects of the pathological cascade in NPC1 are lacking. However, recently an increase in amyloid beta (specifically Aβ42) and total tau was reported in CSF from NPC1 patients [28]. Furthermore, non-enzymatic cholesterol oxidation products have also been correlated with disease progression and severity [31], [32]. In the current study, we investigate protein expression changes that occur in pre-symptomatic *Npc1* mutant mice to acquire a set of potential protein biomarkers related to early pathological processes in NPC1. We further extend these initial studies to a select group of proteins to establish altered levels in NPC1 patients therefore validating the clinical utility of these protein biomarkers. Biomarkers corresponding to multiple aspects of the pathological cascade will provide the tools necessary for the development of therapeutic treatments targeting these secondary consequences.

## Results

### Protein Identification and Validation

**Figure 1:**
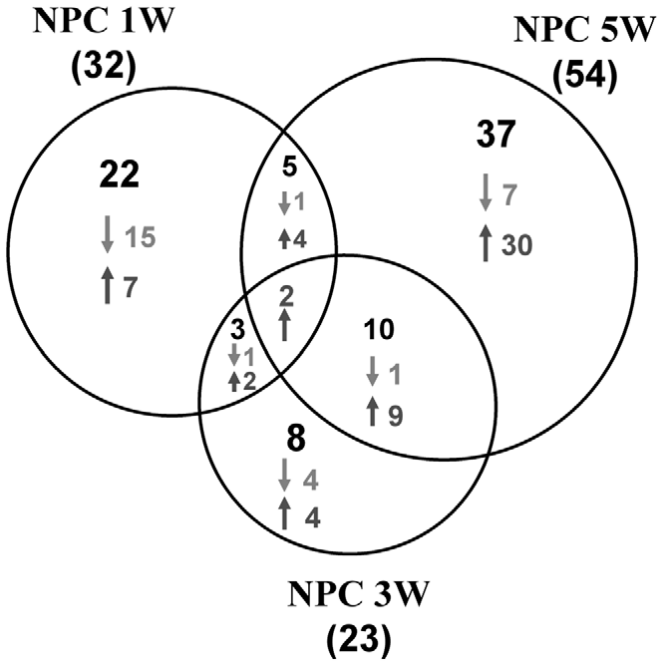
Summary of 2D-GE Differential Protein Spots. Venn diagram depicting gel spots that were identified at each time point. Numbers noted in parenthesis are the total number spots with differential intensity identified at that specific time point. Within each time point the breakdown of spots is provided in which the gel intensity suggested either increased (up arrow) or decreased (down arrow) expression relative to the control. A total of 109 spots were differentially expressed. In week one, 22 were unique to this time point whereas 8 were unique to week three and 37 were unique to week five. From the MS analysis, a total of 77 unique proteins were identified in both the mutant and control (paired) gel spots. The protein identifications included 49 increased and 22 decreased.

In this work, we investigated protein expression changes observed in cerebella collected from 1, 3 and 5 week mutant (*Npc1* ^−/−^) and control (*Npc1^+/+^*) female mice using two dimensional gel electrophoresis (2D-GE) and mass spectrometry (MS) as a discovery tool. Representative silver stained 2D-GE images are provided for control and mutant cerebellum pooled protein samples at each of the time points analyzed (Figure S1). The criteria for selecting differentially expressed gel spots for further analysis was determined based on our previous work which showed a minimal false discovery rate when spot intensity ratios were: R>1.5, R<0.67 (p<0.05) [33]. From the 2D-GE analysis, a total of 109 gel spots were differentially expressed (77 increased and 32 decreased) as is shown in the Venn diagram in Figure 1. From the 109 gel spots, we identified 77 unique proteins, in both the mutant and control samples from MS-based analysis, and a list of these proteins and their quantitative data is provided in Table S1. The 77 protein identifications included 49 proteins which were increased and 22 decreased. For 6 proteins, both increased spots and decreased spots were observed at the same time point or at different time points suggesting differences in posttranslational modification status. Interestingly, only 2 spots identified were found to have altered expression at all three time points. These two gel spots were found to contain transferrin and charged multivesicular protein 2A. Of the differentially expressed proteins, fatty acid binding protein 3, transferrin, transthyretin, glutathione S-transferase P1 (GSTP1), charged multivesicular body protein 2a (CHM2A), 3-phosphoglycerate dehydrogenase (3-PGDH), proteasome subunit alpha, type 1 (PMSA1) and voltage dependent anion-selective channel protein 2 (VDAC2) were chosen to be validated via western blot analysis. Of the proteins chosen, we successfully validated differential expression for at least one time point for six of the eight proteins (75%, see Figure S3). The two proteins in which we were unable to confirm differential expression obtained from the proteomics data were VDAC2 and 3-PGDH. It is important to note that VDAC2 was identified in more than one gel spot from 2D-GE and MS analysis; therefore, differential expression of a particular form separated via 2D-GE would not necessarily be detectable in a one-dimension SDS-PAGE gel separation [34]. Secondly, 3-PGDH western blot analysis showed two bands in which we were unable to definitively distinguish which band corresponds to the differentially expressed protein. Both VDAC2 and 3PGDH have reported post-translational modifications (www.uniprot.org) which may interfere with the validation. An additional complication is the potential cross-reactivity of the VDAC2 antibody to other isoforms such as VDAC1 and VDAC3. Thus there are multiple explanations why validation by western blot might not be accurate. These results provide a conservative estimate of the accurate identification rate of differentially identified proteins.

**Figure 2:**
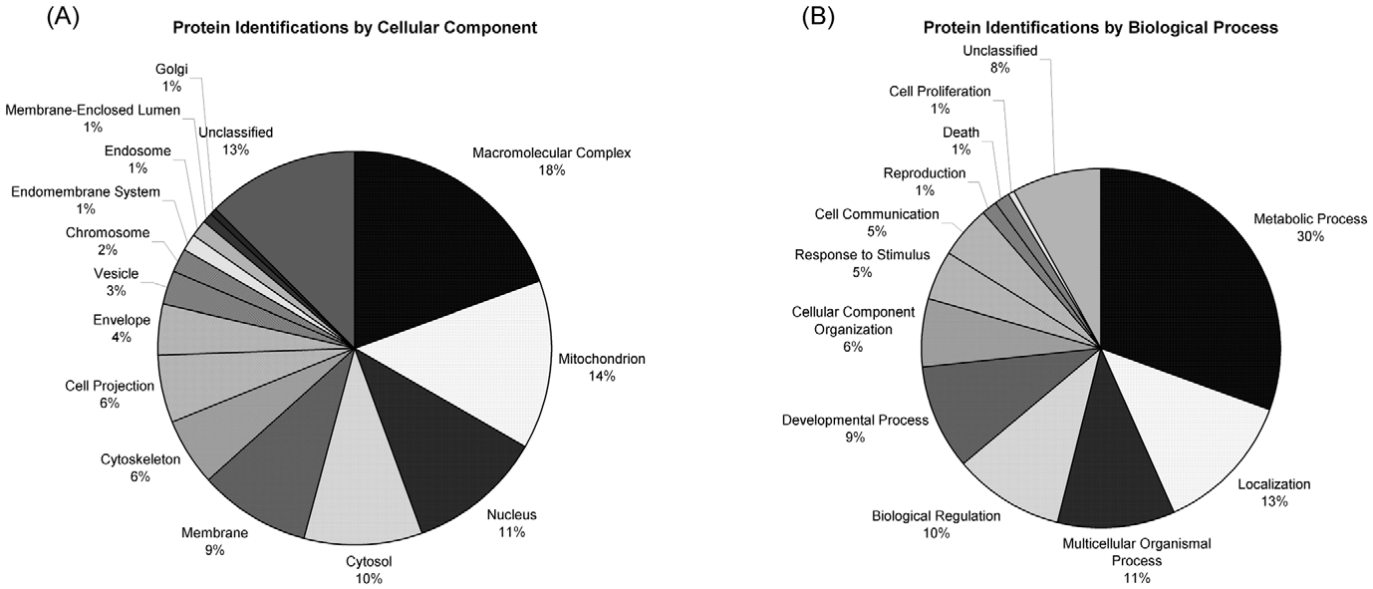
Protein Characterization. Distribution of differentially expressed proteins by cellular location (A), and biological process (B).

Using the protein identifications obtained from the MS analysis, we evaluated the distribution of proteins with respect to cellular location, molecular function and biological process. The top ranking cellular locations (>10 proteins) included macromolecular complexes, mitochondrion, nucleus, cytosol and membrane (Figure 2A). We did not observe proteins specifically located in lysosomes in this study; however, a number of proteins involved in proton pumping were found to be altered. The major biological alterations included metabolic, localization, and multicellular organismal processes (Figure 2B). The categorization of proteins by molecular function was also obtained and is provided in Figure S2. It is important to note that in some cases more than one protein was identified in the same gel spot. For example, triosephosphate isomerase I and glutathione S-transferase Mu 5 were identified in the same spot in the control and *Npc1^−/−^* samples therefore, based on this dataset, we are unable to distinguish whether only one protein is decreased at week five or if both are decreased. Furthermore, at the one week time point, ATP Synthase-beta was identified in the same spot as tubulin beta-2B. However at the five week time point, ATP Synthase was the only identified protein. Additional glycolytic or tricarboxylic acid enzymes that were identified in a spot with secondary confident protein identification included 6-phosphofructokinase, malate dehydrogenase (cytosolic and mitochondrial) and glyceraldehyde-3-phosphase dehydrogenase. As a note, one of the spots in which malate dehydrogenase (mitochondrial) was identified glyceraldehyde-3-phosphate dehydrogenase was also found, therefore, our confidence is increased in that the expression of one of the two enzymes is being perturbed.

**Kyoto Encyclopedia of Genes and Genomes (KEGG)** pathway analysis was used to identify biological pathways with proteins of altered expression. The ten pathways with the lowest p-values are shown in Table 1. Based on the KEGG pathway analysis, several intriguing and previously unsuspected processes appear disturbed in NPC1. These include an apparent disruption of carbohydrate metabolism, with identification of metabolic pathways associated with pyruvate metabolism (p = 3.3×10^−8^), glycolysis (p = 5.5×10^−8^), citrate cycle (p = 4.6×10^−7^) and fructose/mannose metabolism (p = 5.4×10^−5^). KEGG analysis also identified an alteration of proteins associated with Alzheimer disease (p = 7.3×10^−5^). Alzheimer disease is a common neurodegenerative disorder with some pathological overlap with NPC1 [28]. An alteration of glutathione metabolism (p = 3.0×10^−6^) was also identified. This is consistent with increased oxidative stress in NCP1 [9], [11], [13], and also explains identification of pathways associated with drug and xenobiotic metabolism. We evaluated a few of these pathways in greater depth, and a detailed list of the identified proteins categorized by pathway is provided in Table S2. It important to note that while the primary defect of NPC1 causes a secondary disruption in cholesterol biosynthesis, we did not identify proteins from this pathway possibly due to the fact that many are membrane proteins and our 2D-GE method was not optimized for membrane protein identification.

**Table 1:** KEGG Pathway Analysis of Differentially Expressed Proteins.

Enriched KEGG Pathway	No. of Proteins	Adjusted p-value
Pyruvate Metabolism	5	3.34E-08
Glycolysis	6	5.47E-08
Citrate Cycle	4	4.63E-07
Proteasome	4	2.26E-06
Glutathione Metabolism	4	3.04E-06
Fructose and Mannose Metabolism	3	5.39E-05
Alzheimer Disease	5	7.34E-05
Metabolism of Xenobiotics by Cytochrome P450	3	3.00E-04
Drug Metabolism - Cytochrome P450	3	4.00E-04

KEGG pathway analysis of the top ten significant pathways altered in the *Npc1^−/−^* mouse cerebella. The pathway along with the number of proteins associated with the pathway and the p-value are provided.

### Glucose Metabolism

Several proteins associated with glycolysis were identified in our study. KEGG pathway analysis indicated significant alterations of proteins involved glucose catabolism including glycolysis, pryuvate metabolism and the citric acid cycle. The altered glycolytic enzymes responsible for conversion of glucose to pyruvate identified were: 6– phosphofructokinase, triosephosphate isomerase I, and pyruvate kinase isozymes M1/M2. The statistically significant differences for these proteins showed decreased expression with mutant to control ratios of 0.47 (p = 0.03, week 3), 0.41 (p = 0.03 week 5) and 0.46 (p = 0.05, week 1), respectively. Breakdown of pyruvate generated from glycolysis to acetyl CoA provides the link between glycolysis and the citric acid cycle. Pyruvate dehydrogenase (E1 component of the beta subunit), is responsible for the conversion of pyruvate to acetyl CoA, (via oxidative decarboxylation) and was found to be significantly decreased at the 1 week time point (R = 0.60, p = 0.03).

Enzymes involved in the citric acid cycle were also found to be altered. Two enzymes found to be decreased only at the week 1 time point were isocitrate dehydrogenase (R = 0.46, p = 0.02) and ATP synthase–beta (R = 0.59, p = 0.01). Interestingly, ATP synthase-beta was identified as increased in the 5 week time point (R = 1.57, p = 0.04) however shifted on the 2D gel to higher molecular weight and lower isoelectric point. This protein has been reported to be both acetylated [35] and phosphorylated [36]; therefore, post-translational modification could potentially explain this shift. Malate dehydrogenase (mitochondrial form), which is responsible for the conversion of malate to oxaloacetate and subsequent NADH release, increased at the week three time point (R = 1.87, p = 0.02). Furthermore, the mitochondrial form of malate dehydrogenase was also identified in an adjacent spot with significant differential expression at the five week time point (R = 0.54, p = 0.03). In addition to the mitochondrial form, the cytosolic form of malate dehydrogenase was also identified to be differentially expressed at five weeks (R = 2.12, p = 0.03).

**Figure 3:**
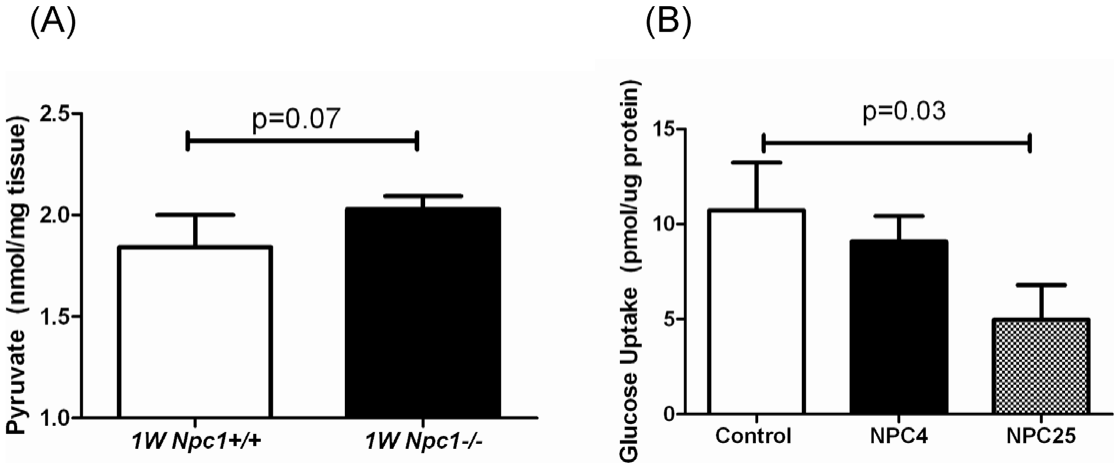
Glucose Metabolism Studies. (A) Measurement of pryuvate concentrations in control (mean = 1.84±0.16 nmol/mg) and 1 week mutant mice (mean = 2.03±0.06 nmol/mg) cerebella (p = 0.07, n = 4). (B) Glucose utilization assay using cultured fibroblasts from Niemann-Pick disease, type C1 patients and control (three replicates). The mean values were 10.72±2.52, 9.08±1.33, and 4.96±1.82 pmol/µg for control, NPC4 and NPC25, respectively. NPC4 (homozygous I1061T, severity score 14) represents a typical presentation of the disease whereas NPC25 (c.2979dupA, N701K, severity score 40) represents a severe presentation. Data are represented as average ± standard deviation. Significance was calculated using an unpaired t-test.

The above proteomic findings suggest that there may be a defect in glucose metabolism in NPC1. Thus we conducted a series of experiments to determine if we could identify a functional defect of glucose metabolism. First we measured pyruvate levels in *Npc1* mouse cerebellar tissue lysates. Normalized pyruvate levels trend higher (p = 0.07) in the 1 week *Npc1* mutant cerebella relative to controls (Figure 3A). Second, we measured glucose uptake in human fibroblasts isolated from control, a typical NPC1 patient (NPC4, homozygous I1061T, severity score 14) and a severely affected child (NPC25, c.2979dupA, N701K, severity score 40). The uptake of 2-deoxy-D-[1,2-^3^H]-glucose was significantly lower (mean and standard deviation = 4.96±1.82 pmol/µg, p = 0.03, Figure 3B) in the NPC25 cell line compared to control values (mean and standard deviation = 10.72±2.52, pmol/µg). Glucose uptake in the NPC4 cell line was intermediate between control and NPC25.

The serine biosynthetic pathway stems from glycolysis. The enzyme 3-phosphoglycerate dehydrogenase (3PGDH) catalyzes the first step of serine biosynthesis and was found to be significantly decreased in the week 1 tissue (R = 0.41, p = 0.05) and continued to be decreased; however, not significantly at the week 3 (R = 0.84, p = 0.27) and week 5 (R = 0.78, P = 0.48) time points. In the central nervous system 3PGDH shunts 3-phosphoglycerate from the glycolytic pathway to yield 3-phosphohydroxypyruvate. 2-phosphohydroxypyruvate is then metabolized sequentially to yield 3-phosphoserine and serine. Glycine can be synthesized from serine by the action of serine hydroxymethyl transferase. Mutations impairing 3PGDH function result in altered serine and glycine levels in the central nervous system [37]. Given the potential of decreased glycolysis combined with a deficiency of 3PGDH, we investigated if serine and glycine levels were altered in the CSF of 12 NPC1 patients (Figure S4). Mean values of both serine (37.4 µM (0.5–4 yr), 29.2 µM (4–14 yr) and 26.6 µM (15+ yr)) and glycine (3.4 µM (0.5–4 yr), 4.0 µM (4–14 yr) and 5.9 µM (15+ yr)) were within normal pediatric reference ranges.

### Glutathione Metabolism and Associations with Oxidative Stress

**Figure 4:**
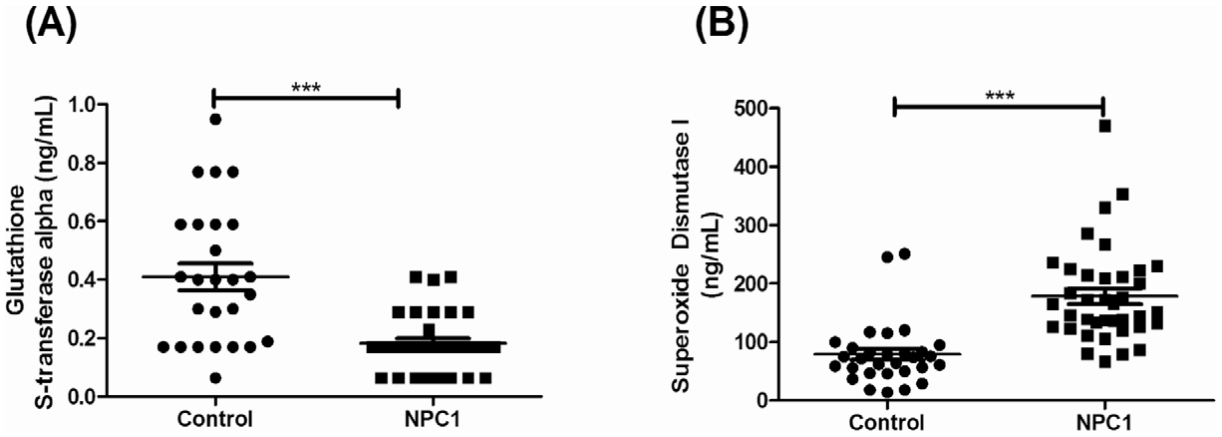
CSF Oxidative Stress Protein Measurements. ELISA-based quantification of oxidative stress-related proteins glutathione-s transferase alpha (A) and superoxide dismutase 1 (soluble) (B) in CSF of Niemann-Pick disease, type C patients and controls. Data are represented as average ± standard error of the mean. Significance is p<0.0001 (***) was determined using an unpaired t-test with Welch's correction. Total number of samples were N = 26 (control) and N = 33 (NPC1) for GSTα. For superoxide dismutase data is represented for N = 30 (control) and N = 38 (NPC1) samples. Values for 4 control and 5 NPC1 patients in the GSTα graph were excluded as they were deemed below the limit of detection of the assay.

Based on KEGG pathway analysis, a series of detoxification enzymes associated with glutathione metabolism and likely oxidative stress were identified to be differentially expressed in the *Npc1^−/−^* cerebellar tissue lysate relative to control. Glutathione S-transferase (GST) mu 5 (R = 0.41, p = 0.03, week 5), glutathione S-transferase alpha 4 (R = 1.83, p = 0.03, week 1), glutathione S-transferase, pi 1 (R = 2.18, p = 0.01, week 1) and isocitrate dehydrogenase 2 (NADP+), mitochondrial (R = 0.46, p = 0.02, week 1) are included in this pathway. To further investigate possible oxidative stress-related protein expression in NPC1 patients, we measured the alpha-family of GSTs in CSF relative to controls and observed a significant decrease (p<0.0001) (Figure 4A). The average control CSF GST-alpha concentration was 0.41 ng/mL (±0.05) compared to NPC1 patients which had an average concentration of 0.18 ng/mL (±0.02). Additionally, cytoplasmic superoxide dismutase 1-soluble (SOD1) was measured in the CSF of NPC1 patients in which a significant (p<0.0001) increase was observed in NPC1 patients (Figure 4B). The SOD1 concentrations were 79.1 ng/mL (±9.8) for controls and 178.1 ng/mL (±13.3) for NPC1 patients. We did not observe a correlation of SOD1 or GST-alpha concentration with respect to patient age, disease severity or treatment with miglustat (data not shown).

### Alzheimer Disease-Associated Proteins

The proteins identified in this study to be differentially expressed and related to Alzheimer disease include: glyceraldehyde-3-phosphate dehydrogenase (GAPDH), cyclin-dependent kinase 5 (CDK5), ATP synthase-beta (ATPB), microtubule-associated protein tau (TAU) and protein phosphatase 3 – alpha (PPP3CA). GAPDH was identified in three gel spots at the five week time points with ratios of 2.21 (p = 0.05), 1.83 (p = 0.05), and 0.54 (p = 0.03) indicative of a highly modified protein. CDK5 was found to be increased at the 1 week time point with a ratio of 2.03 (p = 0.02). ATPB was also increased at the 5 week time point with a mutant to control ratio of 1.57 (p = 0.04). TAU was found to be decreased at the one week time point (R = 0.33, p = 0.03) however it should be noted that two other proteins were identified in this same gel band. Finally, protein phosphatase 3 was found to be decreased in the *Npc1^−/−^* tissue at both the one and three week time points where the mutant to control ratios were 0.046 (p = 0.01) and R = 0.66 (p = 0.01), respectively.

Further analysis with respect to correlations between NPC1 and AD was performed via an Alzheimer mRNA expression analysis (Table S3). Post-mortem tissue (cerebellum and frontal cortex) from three control and three NPC1 patients was used for the analysis. From the AD array data, the CDK5 transcript was found to be decreased in the NPC1 human frontal cortex (fold change: −1.41, p = 0.37) and cerebellum (fold change: −1.67, p = 0.09). TAU was not found to be significantly altered in either the frontal cortex (fold change: 1.08, p = 0.68) or the cerebellum (fold change: 1.33, p = 0.38) in human NPC1 tissue relative to control. Changes in GAPDH were also non-significant in either tissue (frontal cortex fold change: 1.07, p = 0.63, and cerebellum fold change: 1.23, p = 0.61). The AD array results revealed significant increased expression of SERPINA3 (α-1-antichymotrypsin). SERPINA3 mRNA was found to be increased in the human NPC1 cortex 62-fold (p = 0.04) and 16-fold (p = 0.02) in the cerebellum compared to control tissue. Further validation of this differential expression was carried out via western blot on the human frontal cortex tissue and human cerebellum in which an increase in protein expression was also observed (Figure S5). An additional member of the serpin protein family was found in our proteomics study, leukocyte elastase inhibitor, a (Serpin B1a) which was elevated at the three week time point (R = 2.16, p = 0.02).

### Charged multivesicular body protein 2a

**Figure 5:**
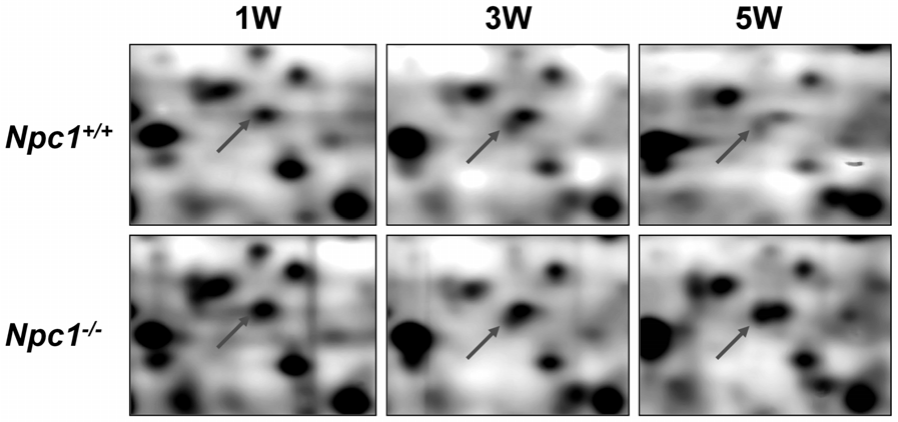
2D-GE Image of CHM2A Protein Spot. Representative 2D-GE images of CHM2A expression over the time course study. Arrows indicate the protein spot of interest. Spots for each genotype and at each time point represent triplicate analysis of a protein pool comprised of n = 4 to 6 cerebella lysates.

Charged multivesicular body protein 2a (CHM2A) also known as chromatin-modifying protein 2a was found to be over-expressed in the mutant relative to control at all three time points in this study. At one week of age, the mutant to control ratio was 1.62 (p = 0.01) while at three weeks the ratio was 1.52 (p = 0.01) and the week five ratio was 2.74 (p = 0.04). Representative gel images of the CHM2A spots across the time course study are provided in Figure 5. MS-analysis confirmed the identity of this protein at the one and three week time points. CHM2A is a small coiled-coil cytosolic protein which has been shown to bind the suppressor of potassium transport growth defect 1 protein and is involved in vacuolar protein sorting [38]. Moreover, CHM2A is a component of the ESCRT (endosomal sorting complexes required for transport) -III complex which is involved in the formation of multivesicular bodies (MVB) in the late endosomal compartment that are responsible for ubiquitin-dependent degradation [39]. The increase in CHM2A expression may be attributed to an increase in the number of MVBs in NPC1. An increase in the size and number of MVBs has been previously reported in Alzheimer disease [40]. Consistent with an alteration in ubiquitin-dependent degradation, we observed several proteins associated with the proteasome to be increased at the five week time point. These include; proteasome activator complex subunit 3, (R = 1.90, p = 0.05), 26S proteasome non-ATPase regulatory subunit 8, (R = 3.44, p = 0.04), proteasome subunit beta type-7, (R = 1.75, p = 0.05) and proteasome subunit alpha type-1, (R = 3.55, p = 0.003). Increased expression of proteins that support this macromolecular structure and function suggests an increase in protein degradation in NPC1.

### Fatty Acid Binding Proteins

**Figure 6:**
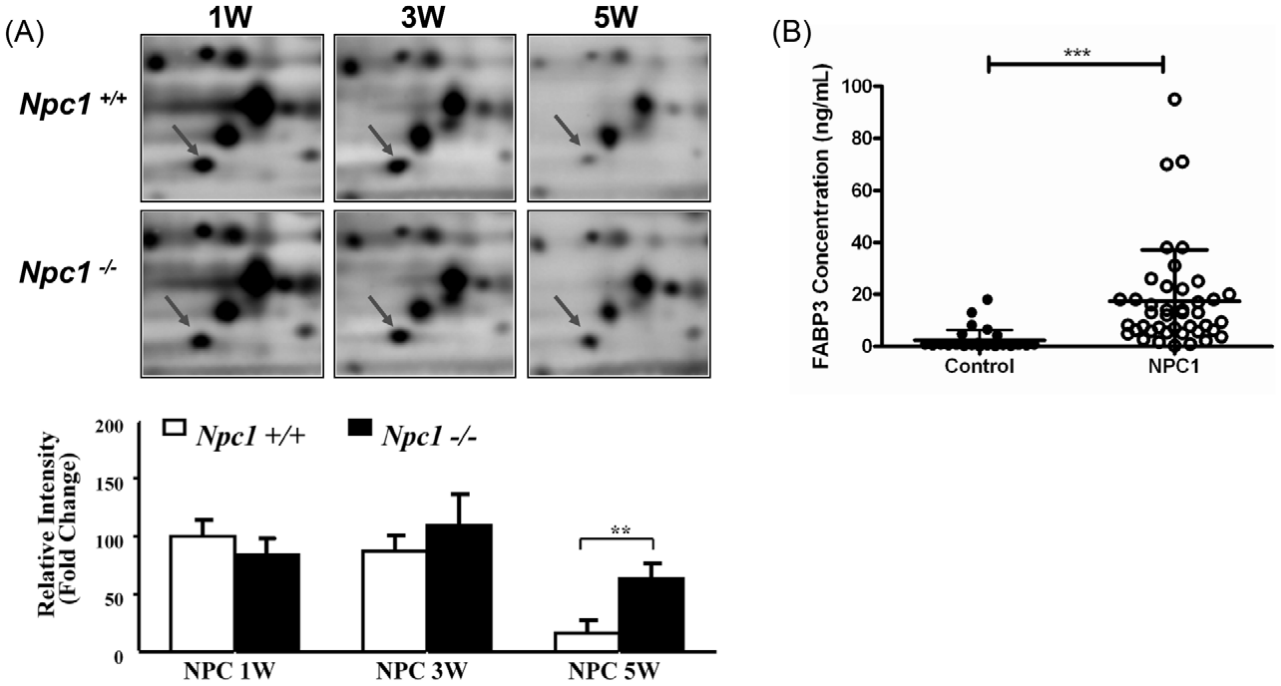
Expression of fatty acid binding protein 3 (FABP3). (A) Representative 2D-GE images of FABP3 showing the over-expression in the mutant mouse cerebellum compared to the control tissue at the week five time point (top) and graphically displayed (bottom). Arrows note the protein spot of interest. (B) FABP3 levels measured in CSF from control (n = 30) and NPC1 patients (n = 42). Data is represented as average ± standard error of the mean. Significance was determined using an unpaired t-test with Welch's correction, p<0.0001.

Fatty acid binding protein 3 (FABP3) was increased at the five week time point in the *Npc1^−/−^* tissue relative to control (R = 3.90, p = 0.01). Representative images of the gel spot corresponding to FABP3 and the quantitative spot intensities over the entire time course from the 2D-GE analysis are provided in Figure 6A. From the image, it appears as though a down-regulation occurs in the control animal lysate which does not occur in the mutant lysate during development. Taqman® gene expression analysis displayed high expression levels at 1 week of age followed by a decrease at 3 and 5 weeks in control animals (Figure S6). There was not an increase in *Fabp3* gene expression at 5 weeks in the *Npc1^−/−^* mouse cerebellar tissue. This result suggests that the increase in gel spot intensity for FABP3 reflects a post-translational modification of the protein and that the transcript levels are not altered between control and mutant animals. Interestingly, in the proteomics data, differentially expressed FABP3 at the five week time point (R = 1.75, p = 0.03) was also identified in an additional spot from the gel however at a more acidic pI value but of the approximate same molecular weight. This finding suggests that two forms of FABP3 are both increased relative to the control. Only a single phosphorylation site has been reported in the Uniprot Database (www.uniprot.org). Based on manual inspection of the tryptic peptides observed in the mass spectra from this gel spot, we were unable to confirm a modification of the FABP3 protein. Two other members of the FABP family were also found to be differentially expressed in cerebellar brain tissue. These include FABP5 and FABP7. At the week 5 time point, the FABP5 cerebellum expression ratio was R = 2.50 (p = 0.01) whereas FABP7 was found to be increased at both the 3 week (R = 1.71, p = 0.04) and the 5 week (R = 2.28, p = 0.02) time points. The FABP family is responsible for binding hydrophobic ligands such as polyunsaturated fatty acids as a transport mechanism. FABP3 is a cytosolic protein that has been shown to preferentially bind omega-6 polyunsaturated fatty acids required for brain development [41].

**Figure 7:**
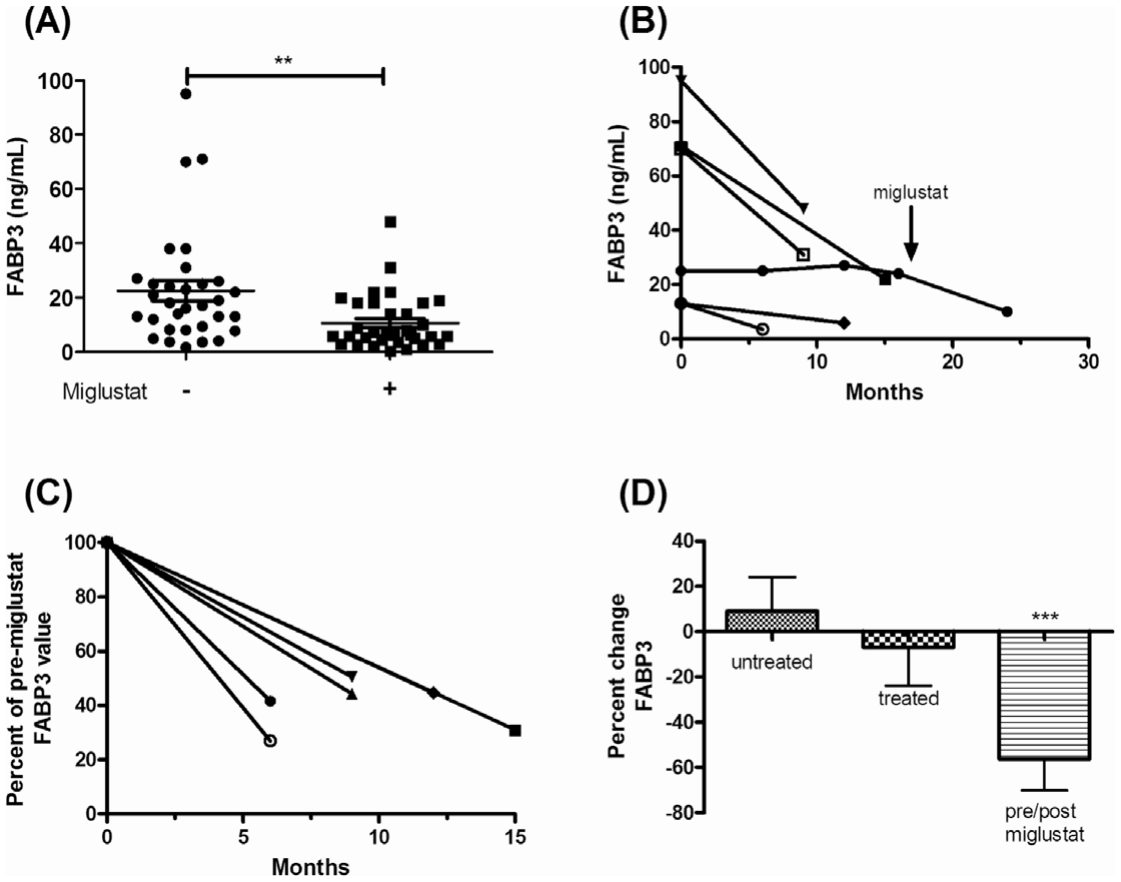
CSF-FABP3 concentration in NPC1 patients and controls. (A) Comparison of FABP3 concentration in untreated (n = 27) and miglustat treated NPC1 patients (n = 18) (p<0.01, t-test, unpaired, Welch's correction). (B) Serial change in FABP3 levels pre- and post-miglustat treatment. Lines connect measurements from the same patient before and after miglustat initiation where one patient was followed serially before miglustat treatment. (C) Percent change in CSF-FABP3 concentration pre- and post-miglustat treatment. (D) Percent change of FABP3 concentration over time in the untreated, treated and pre- post-groups. A one-way ANOVA was used to determined significance (p<0.0001) of the FABP3 concentration change following miglustat initiation.

FABP3 has previously been used as a marker of neuronal damage [42], thus we evaluated CSF levels in NPC1 patients. FABP3 levels in cerebrospinal fluid obtained from NPC1 patients are significantly higher (p<0.0001) than age-matched controls (Figure 6B). The mean FABP3 CSF concentrations for the NPC1 and control cohorts are 17.41 ng/mL (±3.05) and 2.36 ng/mL (±0.72), respectively. Elevation of FABP3 does not correlate with age in either NPC1 patient or pediatric control subjects (data not shown); however FABP3 may be useful to monitor therapeutic interventions. FABP3 values in NPC1 patients on miglustat therapy are significantly lower (p<0.0001) than NPC1 patients not taking miglustat (Figure 7A). This subset of patients was further evaluated by plotting serial values for the patients from whom CSF was collected prior to initiating miglustat therapy and again at least five months after miglustat was started. A decrease in FABP3 levels for these six patients is shown as raw values as well as percent change (Figure 7B–C). When we evaluated FABP3 levels in all serial samples we only observed a significant change in FABP3 levels when the second sample was obtained after initiation of miglustat therapy. In patients untreated with miglustat, over time there was a slight increase (9.0%, Figure 7D) in CSF FABP3 levels. In patients on miglustat therapy at both sampling time points, there was a decrease of 6.9% in CSF FABP3 levels. In contrast, in patients for whom we had CSF samples just prior to initiation of miglustat therapy and subsequent to initiation of therapy, we observed a 56.2% (p<0.001) decrease in FABP3 levels (Figure 7D).

## Discussion

The goals of this study were to (i) to define a set of differentially expressed proteins that can be used as biomarkers to facilitate the development and monitoring of future therapies and (ii) gain a deeper understanding of the early pathological processes perturbed in NPC1. With regards to the first goal, biomarkers can either be disease specific or disease nonspecific. Both have their utility. Biomarkers that are disease specific have the potential to be used as screening or diagnostic tests. Although biomarkers that are not disease specific would not be useful for diagnosis, they can provide information about disease state or progression, and thus be useful in monitoring. The biochemical defect in NPC1 is distinct from other neurodegenerative disorders, but the pathological processes that occur as a consequence of the NPC1 protein deficiency are frequently observed in other neurodegenerative disorders. These include pathological processes such as oxidative stress, neuroinflammation and apoptosis. Protein biomarkers corresponding to these various processes, although not specific for NPC1, are still of great potential utility to monitor disease progression and therapeutic interventions. With regards to the second goal, identification of early pathological processes will lead to a greater understanding of initial events contributing to neurodegeneration and biological pathways that can be targeted for therapeutic intervention.

In this study, we identified altered expression of multiple proteins associated with glucose metabolism. Specifically, KEGG analysis identified alterations in glycolysis, pyruvate metabolism, and citrate cycle. In addition, expression of proteins involved in fructose and mannose metabolism were also altered. A defect in carbohydrate metabolism has not previously been reported. Recent studies have reported abnormalities in brain metabolism specifically hypometabolism in NPC1 patients [43], [44]. Although hypometabolism as ascertained by 5-flourodeoxyglucose is a relatively nonspecific finding in neurodegenerative disorders, it is possible that a defect in carbohydrate metabolism in NPC1 could be a contributing factor to this abnormality. The central nervous system is critically dependent upon glucose metabolism to meet its energy needs, with ketone bodies being the only alternative energy source. We observed a trend toward increased pyruvate levels in cerebellar tissue from *Npc1* mutant mice; however, accumulation of metabolic intermediates does not necessarily occur when multiple enzymes in the same pathway are decreased, but not absent in function. A similar situation occurs in synergistic heterozygosity with metabolism of fatty acids [45]. Multiple defects in a single metabolic pathway decreases flux through the pathway but do not result in a significant accumulation of metabolic intermediates. Functional studies in patient fibroblasts were also consistent with an impairment of glucose utilization, but again the differences were not robust. This could reflect tissue differences (brain versus skin fibroblasts) since sources for energy metabolism are different between these two tissue types or development stages that would not be accurately modeled in vitro. Although additional in vivo studies are needed, the current data suggest that impaired glucose uptake and utilization may be a novel mechanism contributing to the pathology of NPC1.

Recently several groups have reported increased oxidative stress associated with NPC1 [12], [13], [31], [46]. Consistent with these prior studies, our current observation of altered expression of the detoxification enzymes glutathione-s transferase A4 and P1 in mouse cerebellar tissue, combined with our patient data showing altered levels of superoxide dismutase and the alpha-family of GSTs in the CSF of NPC1 patients further supports the hypothesis that oxidative stress may play a role in NPC1 pathology. The discrepant change in tissue glutathione s-transferase α4 and the CSF levels of the entire glutathione s-transferase alpha family may reflect differences between intracellular and extracellular expression. Oxidative stress is a pathological mechanism that occurs in many diseases and neurodegenerative processes. Although treatment of oxidative stress would not correct the primary underlying defect in NPC1, it could provide some benefit especially if considered in the context of treating other aspects of the downstream pathological cascade. The identification of altered superoxide dismutase and glutathione S-transferase in the CSF of NPC1 patients may provide a means to monitor therapeutic impact on this aspect of the disease.

KEGG pathway analysis identified a significant (p<0.0001) alteration in Alzheimer disease related proteins. Specifically, our 2D-GE analysis of cerebellar tissue showed increased and decreased protein expression in *Npc1* mutant mice of CDK5 and TAU, respectively. Gene expression analysis of the human cerebellum tissue showed increased expression of CDK5; however, no change in TAU expression was observed. The apparent discrepancy between the gene and protein expression studies of TAU suggest that altered posttranslational changes may underlie the proteomic result. TAU has been shown to be hyper-phosphorylated in both NPC1 and Alzheimer disease leading to the formation of neurofibrillary tangles (NFTs). Thus, the proteomic data may reflect an alteration in intracellular TAU phosphorylation. Alterations in TAU homeostasis have been observed previously. Nuns *et al.* [47], reported decreased total-TAU and increased phosphorylated-TAU in *Npc1* mutant mouse cerebellar tissue, and Mattsson *et al.* [28] reported increased total-TAU in CSF from NPC1 patients. Of note, total-TAU levels in the NPC1 patients decreased toward control levels in patients treated with miglustat [28]. After identifying an alteration in Alzheimer disease related proteins by KEGG analysis of our proteomic data, we further explored this connection by performing gene expression analysis of control and patient cerebellar and cortical tissue. Of note, we found markedly increased expression of SERPINA3 in cortical (62-fold) and cerebellar (16-fold) tissue from NPC1 patients. Increased protein expression of SERPINA3 was confirmed by western blot. The high levels of SERPINA3 observed in both the human cortex and cerebellar tissue are consistent with the presence of NFTs, as high levels of SERPINA3 and amyloid precursor protein in transgenic AD mouse models display hyperphosphorylation of TAU and tangle formation [48]. Alzheimer disease and NPC1 are distinctly different diseases; however, they, like other secondary tauopathies, share some pathological features. The alternations in TAU homeostasis present in NPC1 provide a potential therapeutic opportunity in that drugs developed for more common disorders that have NFT could have some therapeutic utility in NPC1

We found CHM2A, a member of the ESCRT-III complex which is one of the four complexes of the ESCRT machinery required for cargo transport, and the formation of MVBs [39] to be increased in this study. Note that the ESCRT-III complex functions to promote vesicular budding into the endolysosomal system to form MVBs [49]. MVB's are a prominent histopathological finding in NPC1 [50], [51]. The ESCRT complex has also been shown to participate in ubiquitin-mediated sorting of membrane proteins through the lysosome via E3 ubiquitin ligases and ubiquitination of some membrane proteins may serve as a signaling process [as reviewed in [52]]. SKD1 is a protein required for controlling ESCRT-III assembly and endosomal trafficking that interacts with the ESCRT-III complex to facilitate MVB formation [53], and in low cholesterol environments, NPC1 has been shown to be ubiquitinated and to interact with SKD1 as a sorting signal [54]. While we did not identify any of the E3 ligases in this study, we did identify ubiquitin protein ligase N (R = 3.16, p = 0.03, week 5). Ubiquitin protein ligase N has been shown to act with E3 ligases. We did observe a number of proteasome-related proteins which display differential expression. While this does not fully reveal the mechanisms behind membrane protein sorting and degradation, these results suggest that ubiquitin-mediated processing may be altered in NPC1 disease. Mutations of the *CHMP2B*, a gene encoding a subunit of the ESCRT-III protein, have been identified in some patients with front temporal dementia [55] and amyotrophic lateral sclerosis [56], thus, suggesting that impaired ESCRT-III function can contribute to neurodegenerative disorders. It is not yet clear whether the increased expression of CHM2A that we observed in this study contributes to the pathology of NPC1 or is a secondary consequence of increased MVB formation.

We found increased protein expression of fatty acid binding protein family members in cerebellar tissue from *Npc1* mutant mice. This includes all three members, FABP3, FABP5 and FABP7, of this family known to be expressed in brain tissue, Boneva *et al.* [57], recently described the expression of FABP3, FABP5 and FABP7 in the infantile and adult monkey cerebellum. Both FABP3 and FABP7 were highly expressed in cerebellar Purkinje cells, a neuronal cell type that is very sensitive to the loss of NPC1 protein function. A number of prior studies support the use of FABP as biomarkers in neurological diseases. In adult monkeys, FABP3 and FABP7 are highly expressed in cerebellar tissue; however, FABP5 expression was only observed following ischemic injury [61]. Elevated CSF FABP3 levels have been reported in other neurodegenerative disorders including, Alzheimer disease [58], and Creutzfeldt-Jakob disease [59]. Finally, both FABP3 and FABP7 have been proposed as brain injury markers in serum [42]. The identification of dysregulated expression of FABP3 in cerebellar tissue from *Npc1* mutant mice and identification of elevated levels in CSF from NPC1 patients suggests that FABP3 may have a specific role in the pathology of NPC1. Prior studies have shown that FABP3 functions in both neurite formation and synapse maturation, and may regulate uptake and homeostasis of omega-3 and omega-6 fatty acids. Imbalance of the omega-6/omega-3 fatty acid ratio has been suggested to play a role in the pathogenesis of neurological disorders [60], [61].

Our data, combined with a series of clinical reports and a randomized clinical trial suggesting some efficacy of miglustat therapy in NPC1 [16], [18] begin to validate the use of FABP3 as a surrogate biomarker for treatment of NPC1. The lack of a correlation with increasing patient neurological impairment is not unexpected. CSF levels of FABP3 likely are a reflection of ongoing neuronal cell loss/damage, which occurs throughout the course of the disease, and not an index of remaining neurons. The potential usefulness of monitoring CSF FABP5 and FABP7 levels and the potential to monitor serum levels remains to be explored; however, CSF FABP3 levels provide a tool to help rationally guide the development of an effective treatment of this devastating disorder.

To summarize, this study identifies proteins that are differentially expressed in the *Npc1^−/−^* mouse cerebellum relative to controls, and identifies biological pathways that likely contribute to or are associated with the pathological cascade that leads to neurodegeneration in NPC1 patients. We have identified several proteins and biological pathways that warrant further analysis to identify the exact mechanisms by which they affect the NPC1 phenotype. Several proteins found from our murine studies were further validated in mouse or human tissue, cultured fibroblasts or in the CSF of NPC1 patients. Furthermore, the agreement between FABP3 expression in both the murine model and CSF from NPC1 patients will facilitate validation of this biomarker so that it can be used as a protein marker to assist in the development of therapeutic interventions for this lethal neurodegenerative disorder.

## Materials and Methods

### Ethics Statement

Animal work was performed under an NICHD Animal Care and Use Committee-approved animal study protocol. NPC1 patients included in this study were enrolled between August 2006 and January 2011 in a *Eunice Kennedy Shriver* National Institute of Child Health and Human Development Institutional Review Board -approved longitudinal Natural History/observational trial at the National Institutes of Health (06-CH-0186, NCT00344331). Written informed consent was obtained from the patient, parent or legal guardian. Assent was obtained when appropriate. Consent was witnessed and became part of the patient's medical record at the NIH Clinical Center. The protocol and consent document includes collection of biomaterials and establishment of skin fibroblasts cultures for research purposes.

### Materials

All reagents were used as received unless otherwise stated. Gel electrophoresis materials and chemicals were obtained from Bio-Rad Laboratories (Hercules, CA) or Invitrogen (Carlsbad, CA). HPLC grade acetonitrile and methanol were purchased from Fisher Scientific (Pittsburgh, PA).

### Animal Care and Husbandry

Animal work was performed under the *Eunice Kennedy Shriver* National Institute of Child Health and Human Development Animal Care and Use Committee approved protocol, #12-002. Female *Npc1^−/−^* and *Npc1^+/+^* mice (BALB/cNctr-*Npc1^m1N/m1N^*) were euthanized after 1, 3 and 5 weeks of age, and the cerebella were collected for proteomics studies.

Due to the known gender dimorphism, female mice were used in this study to avoid potentially increasing variability due to gender effects on protein levels. Typically, four to six mice cerebella of each genotype were homogenized and the protein samples were pooled at each time point. Protein concentration was determined using the Bradford Assay and samples were stored at −80°C until analysis.

### Patient Samples

NPC1 patients included in this study were enrolled between August 2006 and January 2011 in an Institutional Review Board-approved longitudinal Natural History observational trial at the National Institutes of Health (06-CH-0186, NCT00344331). Written informed consent was obtained for all subjects as well as assent, when appropriate. The diagnosis was established by biochemical and mutation confirmation of the clinical diagnosis. A total of 42 patients were included. Serial data were available for 15 patients. All NPC1 CSF samples were collected by lumbar puncture in the L4/L5 interspace, after an age-appropriate overnight fast. The lumbar puncture was performed under anesthesia and concurrent with MRI and ABR testing. CSF was collected in a polystyrene tube, and immediately transported to an on-site laboratory where aliquots were stored in polypropylene tubes. Patient identifiers were removed and samples were frozen on dry ice and stored at −80°C prior to being assayed. Control CSF was obtained from 30 gender and age-matched patients who were undergoing CSF collection for another clinical indication. Four control patients were febrile (>38.5°C) at the time of CSF collection, but none had elevated white blood cell count or positive cultures. Age of pediatric control subjects range from two weeks to 20 years at time of CSF collection. Ages of NPC1 patients at the time of CSF collection ranged from 3 months to 52 years. Fibroblasts used in this study were primary cultures derived from 2 mm punch skin biopsies that were cultured in 10% fetal bovine serum (FBS) containing DMEM media.

### Two-dimensional Gel Electrophoresis (2D-GE)

Electrophoresis, staining and imaging was carried out as previously described [33]. 2D-GE was carried out on four gels for each protein pool. Triplicate silver stained gels were analyzed for protein quantification and Coomassie stained gels were prepared for mass spectrometry analysis/protein identification. Spot detection, matching, quantification, normalization and statistical analysis for the triplicate gels were performed in one match set using the PDQuest 8.0 software (Bio-Rad Laboratories) as previously described [33], [62]. A total of 1031 protein spots were matched among the two groups of gels analyzed. Spot intensities were obtained by integration of the Gaussian function with unit of intensity calculated as “intensity x area”. The intensity of each protein spot was normalized using the “total intensity of all spots detected” method to compensate for non-expression related variations in spot quantities between gels. Normalized spot intensity values for the three replicated gels at each time point were analyzed by applying an unpaired Student's t- test (p<0.05). Protein spots considered to be differentially expressed must meet the following criteria: mutant to control ratios of at least 1.5 or 0.67 corresponding to a ±1.5 fold change and p<0.05. Gel imaging data is represented as average ± standard deviation.

### Gel Excision, Protein Digestion and Mass Spectrometry Analysis

Protein spots were excised from preparative gels and digested using trypsin using standard protocols as previously described [33]. Briefly, gel slices were de-stained then dehydrated in a vacuum centrifuge. A total of 200 ng of sequencing grade trypsin (Promega Corp., Madison, WI) was added to the excised gel fragment, covered with 100 mM ammonium bicarbonate and allowed to incubate at 37°C overnight. The supernatant was collected and further extraction was performed by addition of 5% formic acid/ACN (1∶1, v/v) followed by a second extraction step of 5% formic acid/ACN (5∶95, v/v). The combined solutions were vacuum dried and reconstituted in 0.1% TFA followed by concentration and cleanup using C_18_ ZipTips (Millipore). The resulting peptides from the digest were analyzed by MALDI-TOF/TOF using an Applied Biosystems 4800 Proteomics Analyzer and LC-ESI-MS/MS via an LCQ Deca ion trap mass spectrometer (ThermoFisher) equipped with a in-house packed reversed phase column (C_18_ – 75 µm ID x 5 cm, 5 µm particle size) fitted directly at the electrospray source.

Sample preparation for MALDI-MS/MS analysis was as follows. Tryptic peptides were mixed 1∶1 (v/v) with α-cyano-4-hydroxycinnamic acid (5 mg mL^−1^ in ACN/0.1% TFA) and directly spotted onto the MALDI target. MS spectra were calibrated using internal standards and tryptic autolytic peaks. Typically, each MS spectrum was acquired in the positive-ion reflectron mode using 400 laser shots. From each MS spectrum, four to six tryptic peptides were selected for MS/MS analysis. Fragmentation spectra were acquired as unimolecular decompositions (collision gas off) using 1000 laser shots.

LC-ESI-MS/MS data was collected in the data-dependent mode where the three most abundant signals were chosen for isolation and fragmentation. Tandem MS spectra were extracted using BioWorks 2.0 (ThermoFisher).

Database searching was performed by submitting the MS/MS spectra to Mascot v2.2 (Matrix Science) incorporating the *Mus musculus* subset of the SwissProt database (16,365 entries; May 03, 2011). Database searching parameters were as follows: enzyme – trypsin, one missed cleavage, fixed modification – carbamidomethylation (C), variable modification – oxidation (M), precursor mass tolerance - ±0.15 Da (TOF/TOF) and ±1.2 Da (Deca), fragment ion mass tolerance - ±0.06 Da (TOF/TOF) and ±0.6 Da (Deca). Only peptides with individual ion Mowse scores of ≥32 indicating significant identity or extensive homology (p<0.05) were used for protein identification. Scaffold v3.0 (Proteome Software, Portland, OR) was used to validate peptide and protein identifications. Peptide identifications from Mascot were verified using the X! Tandem database search program [63] integrated in the Scaffold software package. Probabilistic validations of peptide identifications were performed using Peptide Prophet [64] and the corresponding protein identifications were determined using Protein Prophet [65]. The Scaffold settings used to validate peptide and protein identifications were as follows: peptide identification: ≥95.0% probability, protein identification: ≥99.0% probability incorporating two or more identified peptides. Proteins identified as differentially expressed were identified in both the mutant and control samples unless otherwise noted. False discovery rates were determined to be ≤0.5% at the peptide level and ≤0.1% at the protein level as calculated using Scaffold.

### Alzheimer Disease Array Analysis and TaqMan Gene Expression

Human cerebellum and frontal cortex tissue from three NPC1 patients and three age-matched controls was obtained from the NICHD Brain and Tissue Bank for Developmental Disorders (University of Maryland, Baltimore, MD). RNA was extracted after homogenization of approximately 100 mg of tissue in TRIzol (Invitrogen, Carlsbad, CA) and was further purified using the Qiagen RNeasy Mini Kit (Qiagen, Valencia, CA). Following extraction, total RNA (1 µg) was reverse-transcribed using the RT^2^ First strand kit (SA Bioscience), as recommended by the manufacturer. The Human Alzheimer Disease RT^2^ Profiler PCR Array (SA Bioscience) was performed on both the human frontal cortex and cerebellum according to the manufacturer's protocol.

Mouse cerebellum tissue was harvested at 1, 3 and 5 weeks post-birth (n = 4). RNA was extracted using TRIzol (Invitrogen, Carlsbad, CA) and was further purified using the Qiagen RNeasy Mini Kit (Qiagen, Valencia, CA). The RNA concentration was determined by measuring the absorbance at 260 nm. Ten micrograms were reverse-transcribed into cDNA using the High Capacity cDNA Archive kit (Applied Biosystems, Framingham, MA). The resulting cDNA was used for the following TaqMan Assays: *Fabp3* (Mm02342494) and *Gapdh* (Rodent GAPDH Control Reagents). Quantitative real-time PCR was performed with an Applied Biosystems 7900 real-time PCR system. Each sample was analyzed in triplicate, using 50 ng of total cDNA for each reaction. qPCR was performed according to the manufacturer's protocol and data was collected and analyzed using the ΔΔCt method [66].

### Western Blotting and Validation

Protein lysates (typically 25–50 µg) were loaded onto a 4–12% gradient NuPAGE Gel (Invitrogen, Carlsbad, CA) and electrophoresis was carried out at 120 V constant voltage. Protein transfer was performed using the iBLOT® dry transfer setup (Invitrogen, Carlsbad, CA) according to the manufacturer's protocol. Following transfer, nitrocellulose membranes were incubated in a blocking buffer followed by incubation with the primary antibody at 4°C overnight. After the initial incubation, the membrane was rinsed and incubated with the appropriate secondary antibody for 1 hour at room temperature. Membrane development was carried out using the Invitrogen WesternBreeze™ chemiluminescence detection kit. Primary antibodies used for validation include; transferrin (1∶2000, Enzo Life Sciences), transthyretin (1∶250, GenWay Biotech), fatty acid binding protein 3 (1∶200, Hycult Biotech), glutathione S transferase P1 (1∶1000, ProteinTech Group), charged multivesicular protein 2a (1∶2000, AbCam), proteasome subunit alpha, type 1 (1∶2000, Santa Cruz Biosciences), voltage dependent anion-selective channel protein 2 (1∶1000, Lifespan Biosciences), 3-phosphoglycerate dehydrogenase, SERPINA3 (1∶2000, AbCam) and actin (1∶10,000, Sigma). For quantitation, band intensities (OD*mm^2^) were normalized to beta-actin using the Quantity One software (Bio-Rad Laboratories, Hercules, CA). Pathway and functional analysis was carried out using WebGestalt with integration to KEGG [67], [68].

### Cell culture, glucose uptake and pyruvate measurements

Glucose uptake experiments were performed on fibroblasts as previously described with minor modifications [69]. Typically 1×10^5^ fibroblasts (passage 6) were adhered to a 6 well plate in DMEM/10%FBS (Invitrogen, Carlsbad, CA) media for 24 hours. The cells were then washed with PBS, exposed to 2-deoxy-D-[1,2-^3^H]-glucose for 10 minutes at 37°C followed by immediate quenching and lysis using RIPA buffer. Total radioactivity was then measured, converted to picomole based on an external calibration curve and normalized to total protein amounts following concentration determined using the BCA assay. Pyruvate measurements were performed using the Pyruvate Assay Kit (Cayman Chemicals, Ann Arbor, MI) according to the manufacturer's protocol and normalized to tissue weight.

### Elisa Protein Validation and Amino Acid Measurements

ELISA-based validation was performed on cerebrospinal fluid from NPC1 patients and age-matched controls using Multi-Analyte Profiling Technology (Rules-Based Medicine, Austin, TX). A CSF analyte value that was below the limit of detection was omitted from presented data. Amino acid concentrations in the CSF from NPC1 patients were measured by Medical Neurogenetics (Atlanta, GA). Statistical calculations were performed using Graph Pad Prism 5.0.

## Supporting Information

Figure S1 Representative silver stained 2D-GE images of cerebellar pooled proteins. (A) 1 week control pool, (B) 1 week mutant pool, (C) 3 week control pool, (D) 3 week mutant pool, (E) 5 week control pool and (F) 5 week mutant pool. Proteins were first separated according to isoelectric point in the first dimension then subsequently by molecular weight in the second dimension.
(TIF)

Figure S2 Categorization of identified differential proteins based on molecular function.
(TIF)

Figure S3 Western blot relative quantification of FABP3 (A), CHM2A (B), GSTP1 (C), TRFE (D), TTR (E) and PSA1 (F). Validation was considered when differential expression at one or more time point was achieved in which the different followed significance (p<0.05) or a trend (p≤0.1). P-values are noted for trends observed. Significance levels are as follows: * (p<0.05) and ** (p<0.01). Beta-actin was used as the loading control for all experiments.
(TIF)

Figure S4 CSF levels of serine (A) and glycine (B) in NPC1 patients. Values are broken out with indicated pediatric reference ranges (dotted line).
(TIF)

Table S1 2D-GE and Protein Identification Data. Spot intensity ratios for each of the time points including p-values are included. Checks indicate protein spots that were excised from the gel whereas those spots that were not excisable are noted with a red x. Sheets that note the protein identification as well as the peptide assignments from each gel spot are also included for each time point.
(XLSX)

Table S2 Significant pathways altered in cerebella from the Npc1 mouse model. For each pathway, the identified protein name and gene symbol are included.
(XLSX)

Table S3 Alzheimer Gene Expression Array. The gene of interest as well as the change in cycle number and fold change with p-values are included for both the analysis of frontal cortex and cerebellum in post-mortem human tissue.
(XLS)
