# Supplementary figures and images for: Quantitative Proteomic Analysis of Niemann-Pick Disease, Type C1 Cerebellum Identifies Protein Biomarkers and Provides Pathological Insight
Source: PLoS One. 2012 Oct 29;7(10):e47845. doi: 10.1371/journal.pone.0047845 (PMC3483225; doi:10.1371/journal.pone.0047845)

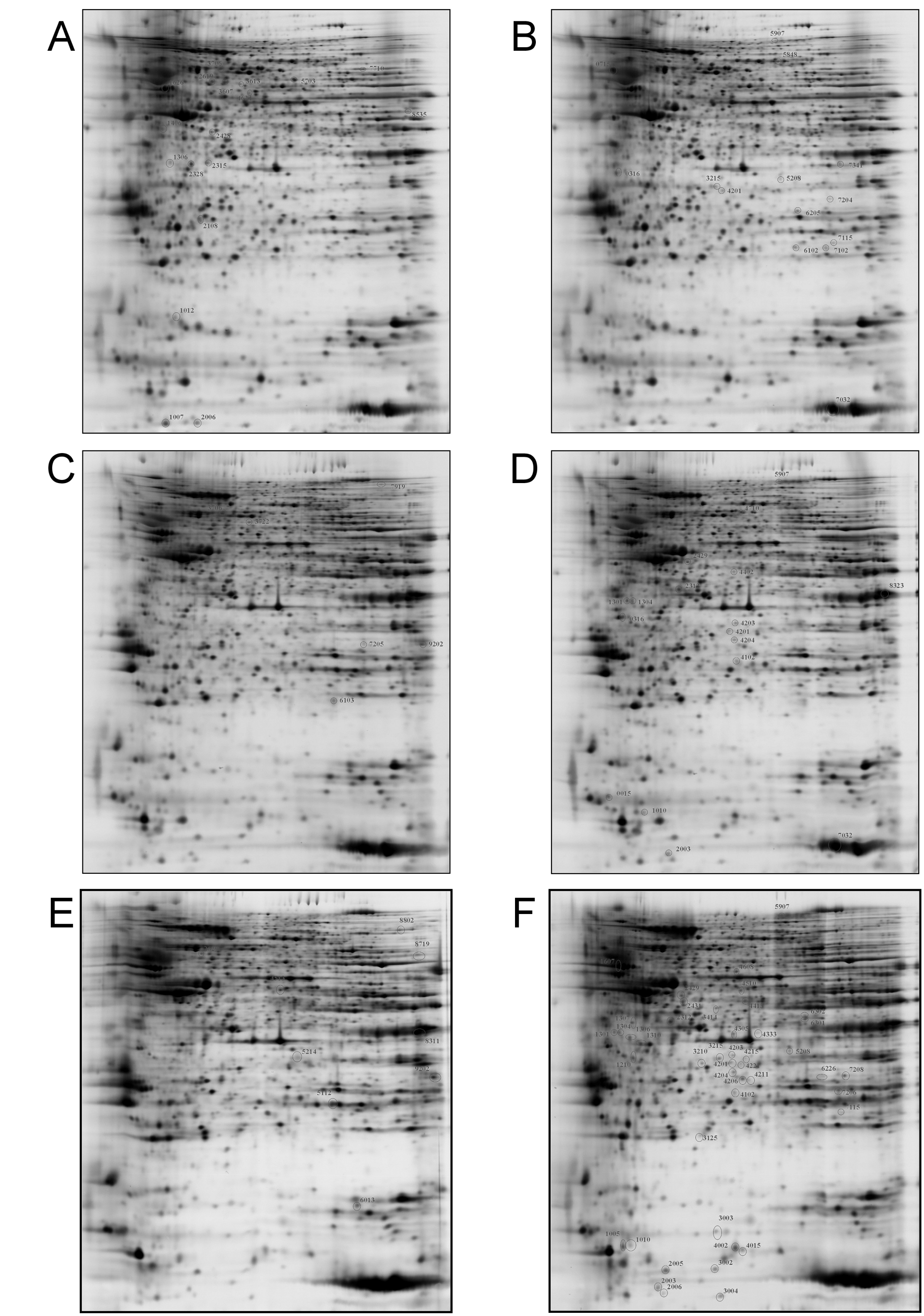

Supplement: Figure S1 — Representative silver stained 2D-GE images of cerebellar pooled proteins. (A) 1 week control pool, (B) 1 week mutant pool, (C) 3 week control pool, (D) 3 week mutant pool, (E) 5 week control pool and (F) 5 week mutant pool. Proteins were first separated according to isoelectric point in the first dimension then subsequently by molecular weight in the second dimension. (TIF) [file pone.0047845.s001.tif]

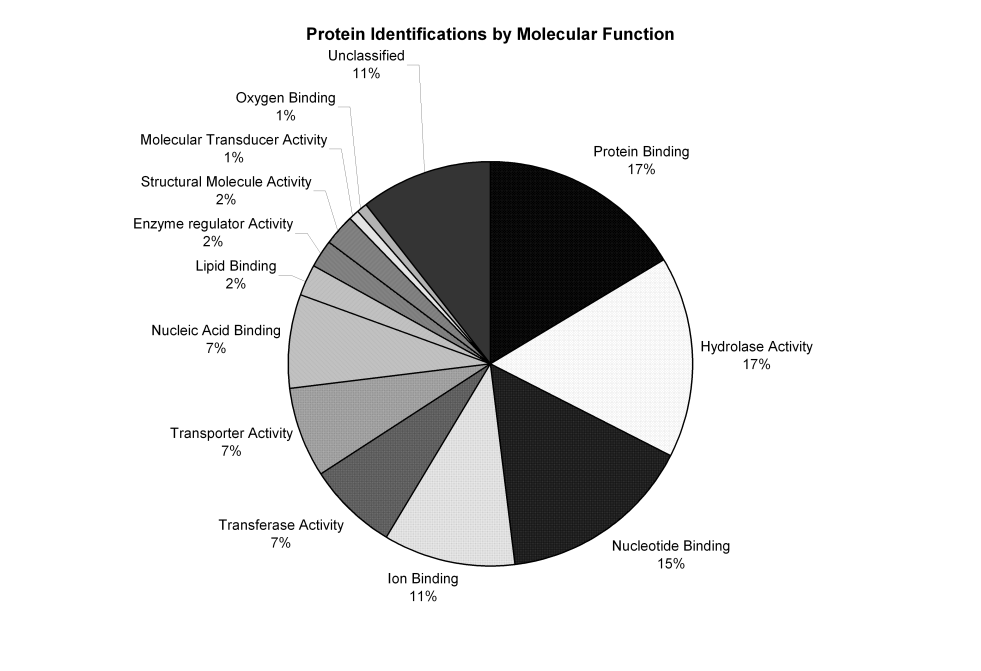

Supplement: Figure S2 — Categorization of identified differential proteins based on molecular function. (TIF) [file pone.0047845.s002.tif]

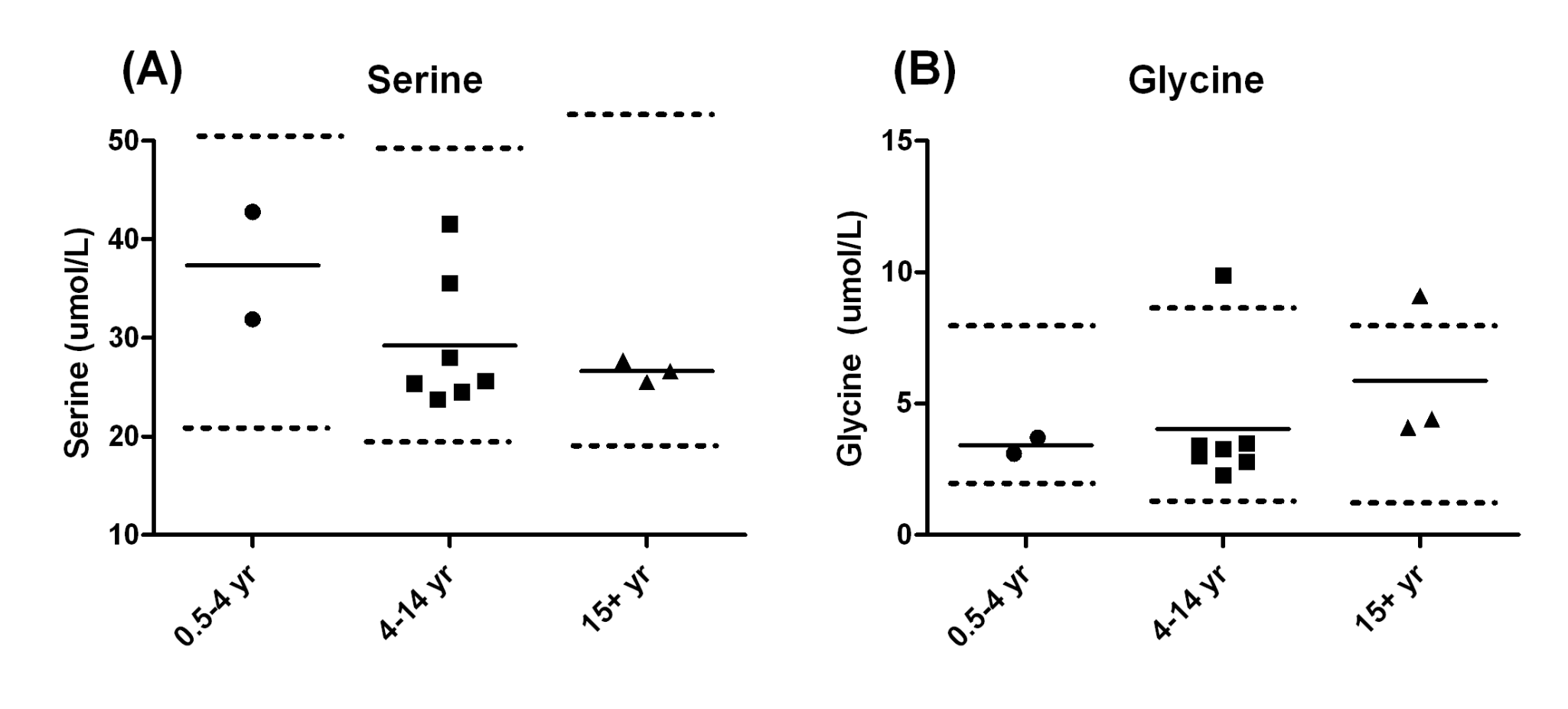

Supplement: Figure S4 — CSF levels of serine (A) and glycine (B) in NPC1 patients. Values are broken out with indicated pediatric reference ranges (dotted line). (TIF) [file pone.0047845.s004.tif]
